# A Method for Sesame (*Sesamum indicum* L.) Organ Segmentation and Phenotypic Parameter Extraction Based on CAVF-PointNet++

**DOI:** 10.3390/plants14182898

**Published:** 2025-09-18

**Authors:** Xinyuan Wei, Qiang Wang, Kaixuan Li, Wuping Zhang

**Affiliations:** College of Software, Shanxi Agricultural University, Jinzhong 030801, China

**Keywords:** sesame, 3D point cloud, CAVF-PointNet++, clustering, organ segmentation, phenotypic extraction, precision agriculture

## Abstract

Efficient and non-destructive extraction of organ-level phenotypic parameters of sesame (*Sesamum indicum* L.) plants is a key bottleneck in current sesame phenotyping research. To address this issue, this study proposes a method for organ segmentation and phenotypic parameter extraction based on CAVF-PointNet++ and geometric clustering. First, this method constructs a high-precision 3D point cloud using multi-view RGB image sequences. Based on the PointNet++ model, a CAVF-PointNet++ model is designed to perform feature learning on point cloud data and realize the automatic segmentation of stems, petioles, and leaves. Meanwhile, different leaves are segmented using curvature-density clustering technology. Based on the results of segmentation, this study extracted a total of six organ-level phenotypic parameters, including plant height, stem diameter, leaf length, leaf width, leaf angle, and leaf area. The experimental results show that in the segmentation tasks of stems, petioles, and leaves, the overall accuracy of CAVF-PointNet++ reaches 96.93%, and the mean intersection over union is 82.56%, which are 1.72% and 3.64% higher than those of PointNet++, demonstrating excellent segmentation performance. Compared with the results of manual segmentation of different leaves, the proposed clustering method achieves high levels in terms of precision, recall, and F1-score, and the segmentation results are highly consistent. In terms of phenotypic parameter measurement, the coefficients of determination between manual measurement values and algorithmic measurement values are 0.984, 0.926, 0.962, 0.942, 0.914, and 0.984 in sequence, with root-mean-square errors of 5.9 cm, 1.24 mm, 1.9 cm, 1.2 cm, 3.5°, and 6.22 cm^2^, respectively. The measurement results of the proposed method show a strong correlation with the actual values, providing strong technical support for sesame phenotyping research and precision agriculture. It is expected to provide reference and support for the automated 3D phenotypic analysis of other crops in the future.

## 1. Introduction

Sesame (*Sesamum indicum* L.), as an important oil crop with both nutritional functions and economic value [1], is highly favored and widely cultivated worldwide. Phenotypic analysis can provide a key basis for screening excellent varieties, optimizing planting schemes, and enhancing plant stress resistance, thereby helping plants achieve high and high-quality yields [2]. Traditional manual measurement of phenotypic parameters is inefficient and prone to data deviation due to subjective judgments. Moreover, it is highly likely to damage the plant’s morphological structure during operation, affecting the accuracy of measurement results and the subsequent growth of plants. Therefore, the development of accurate and non-destructive phenotypic parameter measurement technology plays a crucial role in improving the efficiency of sesame phenotyping, optimizing sesame cultivation and management strategies, and promoting the high-quality development of the sesame industry.

Phenotypic parameter extraction using 2D images offers advantages such as low cost, fast acquisition, and mature algorithms, making it suitable for high-throughput plant phenotyping tasks with simple structures. However, due to the limited information dimension of 2D images, they cannot fully represent 3D morphology; view dependence easily leads to measurement deviations; occlusions make it difficult to obtain information about the blocked parts; lighting conditions affect image quality [3]; and the lack of depth information limits the accuracy of parameter extraction [4]. Ultimately, this may result in significant discrepancies between measurement results and actual phenotypes.

In the field of 3D reconstruction technology, devices such as laser scanners [5], LiDAR [6], professional structured light 3D scanners [7], and TOF cameras [8] can achieve high-precision 3D reconstruction by virtue of their advanced technical principles. However, the purchase cost of these devices is extremely high. For example, a professional-grade laser scanner can cost as much as several hundred thousand CNY or even over a million CNY. Such high costs make them unaffordable for many small and medium-sized enterprises, research institutions, and individual users, thereby limiting their widespread use. Against this backdrop, it is particularly important to find a 3D reconstruction technology with lower cost and stronger adaptability [9]. In contrast, Structure from Motion (SFM) reconstruction technology has significant advantages: it only requires collecting image sequences from different angles using an ordinary camera and then recovers the 3D structure of the object from image feature point matching and motion relationships through algorithms [10], which greatly reduces hardware costs. Meanwhile, this technology has strong adaptability and can efficiently implement 3D reconstruction across scenarios.

The demand for measurement accuracy in plant phenomics has evolved from the plant scale to the organ scale [11]. Segmentation technology is a necessary means to achieve organ-scale analysis [12]. Current mainstream segmentation technologies include clustering-based methods, model-fitting-based methods, and deep learning-based methods. Common characteristics of point clouds include spatial position, color, normal vector, curvature, density, etc. [13]. Clustering-based methods typically utilize one or more of these features to calculate similarity, grouping similar points into categories to achieve segmentation. Miao et al. [14] realized the individual plant segmentation of maize populations through a combined method of Euclidean clustering and K-means clustering. Xiao et al. [15] segmented the soybean canopy and individual leaf point clouds using normal vector differences, an improved region growing algorithm, and curvature features. Ma et al. [16] completed the point cloud segmentation of individual banana plants via the Density-Based Spatial Clustering of Applications with Noise (DBSCAN) clustering algorithm combined with region growing. This type of method achieves good segmentation results for plants with distinct differential characteristics, but the segmentation effect is not ideal when plants are severely adhered or their features are highly similar. Model-fitting-based methods segment regularly shaped organs by matching the corresponding structures of point clouds with preset geometric models. Ao et al. [17] realized the segmentation of individual maize stem instances in the field environment by fitting 3D cylinders. Liang et al. [18] achieved accurate segmentation and localization of bayberry and litchi fruits in complex environments based on a fitting method with approximate spherical shape priors. Choi et al. [19] segmented the fruit parts by fitting ellipsoids to tomato point clouds and estimated the fruit volume by calculating the volume of the ellipsoids. This type of method can accurately segment plants with regular shapes, but it relies on preset models and has poor adaptability to complex organs and occluded scenarios. Deep learning-based methods achieve segmentation by using neural networks to automatically learn the features and spatial relationships of points in point clouds. They then predict category labels point by point. Turgut et al. [20] used a 3D point-based deep learning network, RoseSegNet, to segment the 3D point clouds of rose shrubs into organs such as leaves, stems, and flowers. Song et al. [21] specifically realized the segmentation of cotton organs, including stems, petioles, and leaves, using the deep learning network CotSegNet. Ghahremani et al. [22] achieved the semantic segmentation of spikes and non-spike organs in wheat point cloud data with the deep learning model Pattern-Net. This type of method achieves high accuracy in segmenting complex plants, but it relies on a large amount of manually annotated data for training and also has high requirements for hardware resource configuration.

In recent years, with the rapid development of deep learning technology for 3D point clouds, a variety of emerging network architectures have emerged, further improving the expressive ability and segmentation accuracy of models. LATUP-Net [23] is designed to balance efficiency and accuracy. It extracts multi-scale features through parallel convolutions and introduces an attention mechanism for feature recalibration, which effectively improves segmentation performance while reducing computational overhead. 3D-SCU-Mamba [24] integrates state-space modeling and spatial context modules into its encoder–decoder structure, thereby effectively enhancing global modeling capability and improving training stability. SemiGMMPoint [25] proposes a semi-supervised generative classifier based on the Gaussian Mixture Model (GMM). By optimizing inter-class similarity and adopting a calibrated pseudo-label strategy, it enhances the discriminative ability and robustness of large-scale 3D point cloud segmentation. These methods have significantly improved the accuracy and efficiency of 3D point cloud segmentation by integrating various modeling strategies and learning mechanisms, promoting high-precision and fine-grained analysis in complex scenarios. However, more efficient training strategies and stronger generalization capabilities still need further exploration. This is to meet the demands for large-scale and diverse data in practical applications.

Currently, 3D phenotyping research on major grain crops such as corn, wheat, and soybeans has become relatively mature. However, due to the large number of branches of sesame plants, narrow and long leaves, and their spatial interlacing with stems, clustering-based methods and model-fitting-based methods are difficult to effectively handle such complex topologies, and there is no systematic research so far. This study uses deep learning algorithms to segment the stems, petioles, and leaves of sesame plants. On this basis, geometric clustering technology is applied to complete the task of segmenting leaves from each other.

In plant phenotyping research, the geometric structure of plants can be characterized through morphological traits, and vegetation indices constructed using narrowband or RGB reflectance—such as NDVI [26], false NDVI [27], and PRI [28]—can also be used to reflect the photosynthetic performance and physiological status of plants. Both types of traits are of great significance in crop phenomics. This study focuses more on the morphological characteristics of sesame at the organ scale, so plant height, stem diameter, leaf length, leaf width, leaf angle, and leaf area are selected as core analysis parameters. Plant height reflects the growth rate and vigor of sesame at a specific growth stage and is an important indicator for judging plant robustness. Thicker stems are usually stronger, which not only helps support the plant and reduce the risk of lodging but also has stronger water and nutrient transport capacity, directly affecting the stable growth and fruiting ability of the plant. Leaf length and leaf width together determine the shape and size of leaves, and larger leaves usually have stronger photosynthetic capacity. Leaf angle affects the spatial distribution of leaves and the interception efficiency of light and is an important factor in optimizing canopy structure and improving population photosynthetic performance. The larger the leaf area, the stronger the theoretical photosynthetic potential and the more dry matter accumulation, which is directly related to yield formation. Moreover, these parameters are widely used in phenotyping studies of other crops. For example, Liang [29] et al. extracted parameters such as plant height, stem diameter, leaf area, and leaf angle in their study on tomato seedlings. Sun [30] et al. extracted parameters including plant height, leaf length, leaf width, and leaf area in their research on rapeseed seedling phenotyping. Su [31] et al. extracted parameters like plant height, leaf width, and leaf angle in corn phenotyping studies. Therefore, these parameters not only have cross-crop universality but also can comprehensively reflect the morphological characteristics and growth status of sesame plants.

## 2. Materials and Methods

### 2.1. Image Data Acquisition

The experiment was conducted at the Economic Crop Research Institute of Shanxi Agricultural University in Fenyang City, Lüliang City, Shanxi Province (111°47′ E, 37°16′ N, 827 m above sea level). This area has a temperate continental monsoon climate, with an annual average temperature of approximately 9.8 °C, annual average sunshine duration of 2520 h, an annual average precipitation of about 500 mm, and an annual frost-free period of around 170 days. The soil is mainly loam with medium fertility, which is suitable for the growth of sesame (*Sesamum indicum* L.) and related research.

The sesame varieties tested were Dongbao No.2, Fenzhi No.2, and Fenzhi 13. All three are high-yielding and highly adaptable sesame varieties. Among them, Dongbao No.2 is characterized by stable yield and drought tolerance; Fenzhi No.2 matures early and has a compact plant type; and Fenzhi 13 combines high yield potential with strong stress resistance.

In this study, the field experiment adopted a layout of adjacent variety strips with fixed plots rather than a randomized block design. The experimental field was divided into three adjacent strip-shaped plots, corresponding to the three aforementioned tested varieties for planting, respectively. This experiment was an observational design, focusing mainly on the dynamic changes in phenotypes at different stages rather than strict statistical comparison. This layout facilitates unified management, thereby reducing the interference of management differences on the results and enabling intuitive comparison of the growth performance of different varieties. Each variety was planted in an area of approximately 1500 m^2^, with a row spacing of 1 m and a plant spacing of 20 cm. Each variety had about 7500 plants, totaling approximately 22,500 plants for the three varieties. Sesame seedlings were transplanted on 29 June 2024. After transplantation, watering, fertilization, and pesticide application were carried out regularly according to the growth status of the plants to maintain their healthy growth. To comprehensively capture the phenotypic changes in sesame at different growth stages, point cloud acquisition tests were conducted on the 10th, 20th, 40th, and 60th days after transplantation. For each variety, 3 plants with similar growth vigor were selected as the objects for 3D reconstruction and phenotypic parameter extraction. A total of 9 sesame plants were selected for the entire experiment, resulting in 36 sesame plants with different morphological characteristics across the four time points.

**Figure 1:**
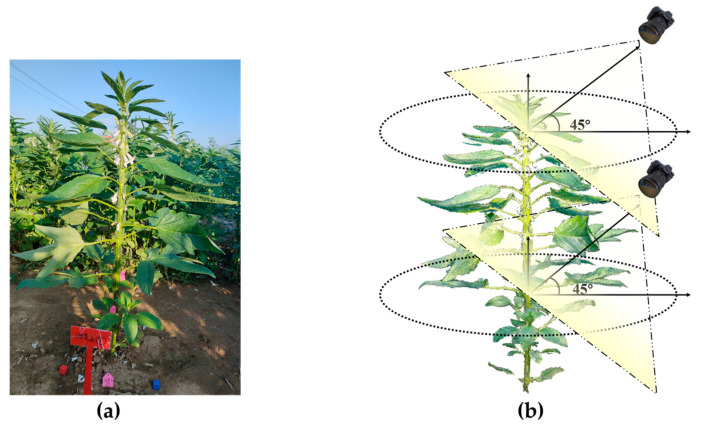
Image acquisition method: (**a**) standard cubic wooden block; (**b**) image acquisition method.

To obtain the spatial scale factor for point cloud reconstruction, a standard cubic wooden block of 2 cm × 2 cm × 2 cm was placed around the sesame plant, as shown in Figure 1a. In this study, RGB images were acquired using a Canon EOS 70D camera (APS-C CMOS sensor, 20.2 MP, resolution 5472 × 3648 pixels, EF lens, Canon Inc., Tokyo, Japan). To ensure consistency in image quality, the camera was set to manual mode with fixed aperture (f/5.6), shutter speed (1/125 s), and ISO (400). Specifically, the white balance was fixed to “daylight mode” (approximately 5500 K) instead of automatic adjustment so as to avoid color shift caused by changes in ambient light. In addition, the shooting time was uniformly scheduled between 5 and 7 a.m. and 17–19 p.m. to avoid strong midday light and the risk of overexposure, thus ensuring consistency in imaging conditions. Image data was captured by surrounding the sesame plant with an RGB camera at a 360° angle. The shooting angle was set to a 45° depression angle, and the distance between the lens and the center of the sesame plant’s canopy was maintained at 30–50 cm to ensure complete coverage of the plant’s morphological characteristics. During the plant’s growth, the distance between the lens and the plant needed to be adjusted in a timely manner according to its growth status, and the shooting angle should be adjusted simultaneously to ensure that the entire plant remained within the frame. As the plant continued to grow, its lower area was highly likely to be occluded. To address this issue, a 360° panoramic shooting method was recommended to fully capture both the upper and lower layers of the plant, as illustrated in Figure 1b. It is crucial to ensure that there is an effective overlapping area between the upper and lower parts of the images during shooting. This overlap can provide a reliable basis for subsequent feature point extraction, thereby facilitating accurate 3D reconstruction of the plant and in-depth analytical research.

### 2.2. Algorithm Overview

**Figure 2:**
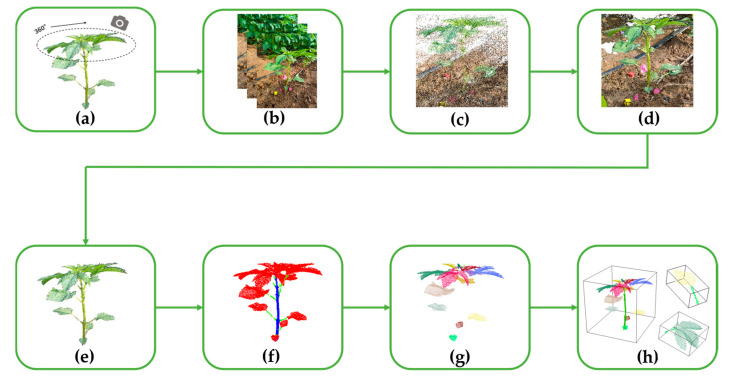
Flowchart diagram of sesame plant reconstruction and phenotypic parameter extraction: (**a**) image data acquisition; (**b**) multi-view image sequence; (**c**) sparse point cloud; (**d**) dense point cloud; (**e**) point cloud preprocessing; (**f**) segmentation of stems, petioles, and leaves; (**g**) segmentation of different leaves; (**h**) phenotypic parameter extraction.

The overall workflow of the method proposed in this study is shown in Figure 2. First, multi-view image data of sesame (*Sesamum indicum* L.) plants at different growth stages were collected using an RGB camera. Second, the 3D point cloud of the plants was constructed using OpenMVG + OpenMVS [32] (version 2.0). Third, after preprocessing, fine annotation of the dataset was completed. A deep model was trained using the CAVF-PointNet++ algorithm based on the annotated data to accomplish the segmentation task of plant stems, petioles, and leaves. Then, the method of curvature-density clustering was combined to realize the segmentation between different leaves. Finally, based on the segmentation results, six phenotypic parameters were extracted, namely plant height, stem diameter, leaf length, leaf width, leaf angle, and leaf area.

### 2.3. Point Cloud Reconstruction and Preprocessing Methods

#### 2.3.1. Point Cloud Reconstruction

During the 3D reconstruction of sesame (*Sesamum indicum* L.) plants, first, the acquired multi-view RGB image sequence is used to construct the 3D point cloud of the plants through OpenMVG + OpenMVS technology.

**Figure 3:**
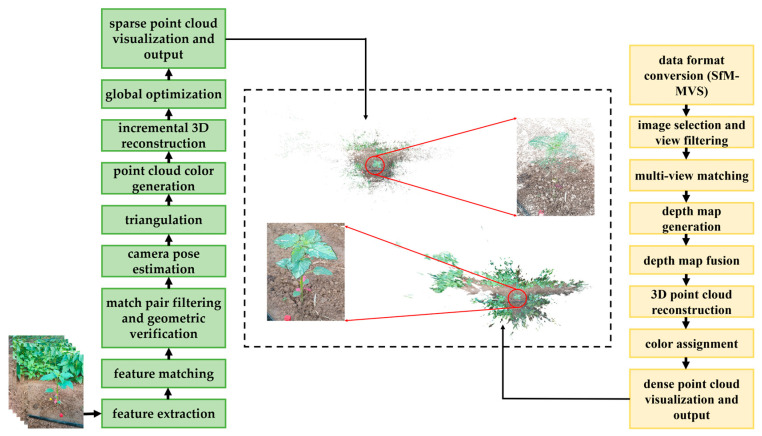
Three-dimensional reconstruction process of sesame plants.

OpenMVG reconstructs the sparse point cloud of the plants through steps such as feature extraction, feature matching, matching pair filtering, and geometric verification; based on the sparse point cloud, OpenMVS further generates a dense point cloud model with higher spatial density and geometric details through steps including image pair selection and view filtering, multi-view matching, and depth map generation. The 3D reconstruction process of sesame plants is shown in Figure 3. This process can effectively restore the 3D structure of sesame plants. The finally generated point cloud file is stored in PLY format, which contains key data such as the coordinate information, color information, and total number of point cloud points, facilitating subsequent analysis and visualization processing.

However, in practical applications, the acquired point cloud has problems with an excessive number of points and the presence of noise points. An excessive number of point cloud points can cause a series of issues, such as reducing computational efficiency, increasing memory usage, and simultaneously posing great difficulties for point cloud visualization work, while the existence of noise points will affect the accuracy of subsequent experiments. To address these problems, it is necessary to adopt a series of filtering operations to effectively solve the aforementioned issues.

#### 2.3.2. Point Cloud Preprocessing

**Figure 4:**
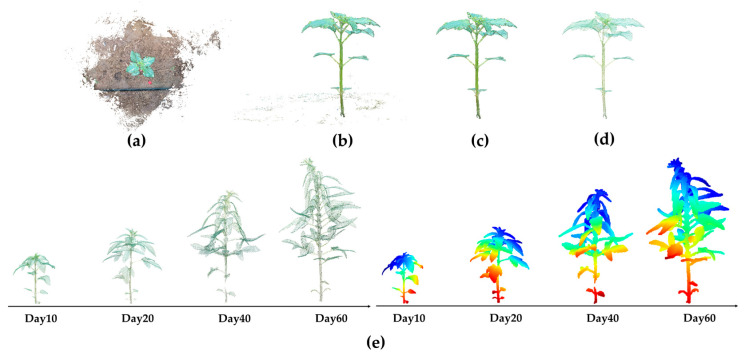
Point cloud preprocessing workflow: (**a**) main structure part; (**b**) color filtering effect; (**c**) statistical filtering effect; (**d**) voxel filtering effect; (**e**) 3D point clouds of sesame plants at different growth stages.

To achieve accurate extraction of the main targets from point cloud reconstruction data, a local density estimation method was adopted to calculate the reciprocal of the average distance of each point within its k neighboring points as a density indicator. A higher density value indicates that the point is located in a region with strong structural continuity and concentrated information. Based on the density distribution characteristics, a reasonable density threshold was set to realize the effective extraction of the main structure part, and the effect is shown in Figure 4a.

Based on the significant color difference between sesame (*Sesamum indicum* L.) plants and the surrounding environment, such as soil—sesame leaves and stems exhibit distinct green characteristics, while the soil and background areas are mainly brown or black—a color feature-based segmentation method was decided to be adopted. This study drew on the filtering idea of the excess green algorithm [33] to achieve segmentation: first, traverse the point cloud data to extract the color components (R, G, B) of each point, then calculate the excess green component value (ExG) of each point to complete the segmentation of plant and environmental point clouds. The calculation formula is shown in Equation (1)

(1) EXG=2G−R−B,

where ExG represents the excess green component value, and R, G, B represent the red, green, and blue color components, respectively.

Considering that the R, G, and B components are susceptible to light intensity and camera exposure, this study minimized the interference of external light changes by fixing camera settings and shooting time periods during the data acquisition phase. In addition, to verify the stability of segmentation, we also tested the normalized excess green index (NExG), whose calculation formula is shown in Equation (2):

(2) $$NExG=\frac{2G-R-B}{R+G+B},$$

Under the conditions of this study, the segmentation effects of ExG and NExG were basically consistent; therefore, the ExG index was mainly used in the subsequent work.

The RGB color component information of the point cloud was viewed using CloudCompare software (Version 2.14.0) [34]. At least 10 points were selected to determine the relationship between the R, G, and B components. Finally, it was determined that points with an ExG value higher than 30 were regarded as the plant part. The effect after color filtering is shown in Figure 4b.

For the existing discrete points, statistical filtering [35] was used for removal. In statistical filtering, the number of neighborhood points (n) and the standard deviation coefficient (α) are key parameters. To obtain the optimal parameters, the results of multiple combinations of n and α parameters were compared. When α was set to 5 and n was 0.8, the filtering algorithm achieved the best balance between noise suppression and structure preservation in denoising the sesame plant point cloud. The filtering effect is shown in Figure 4c.

Finally, voxel filtering [36] was used to downsample the point cloud. When the voxel size was set to 0.04, it could not only effectively reduce the point cloud density and improve computational efficiency but also completely retain the overall morphological structure of the tomato plant point cloud. The effect is shown in Figure 4d. The number of plant point clouds after downsampling is usually maintained between 10,000 and 35,000.

The 3D point clouds of sesame plants on the 10th, 20th, 40th, and 60th days after planting are shown in Figure 4e, and the structural hierarchy and visualization effect were enhanced through Jet color mapping.

#### 2.3.3. Size Conversion

Since there is a certain degree of scaling relationship in size between the constructed 3D point cloud model and the actual sesame (*Sesamum indicum* L.) plant, it is necessary to obtain an accurate scale factor for size conversion.

**Figure 5:**
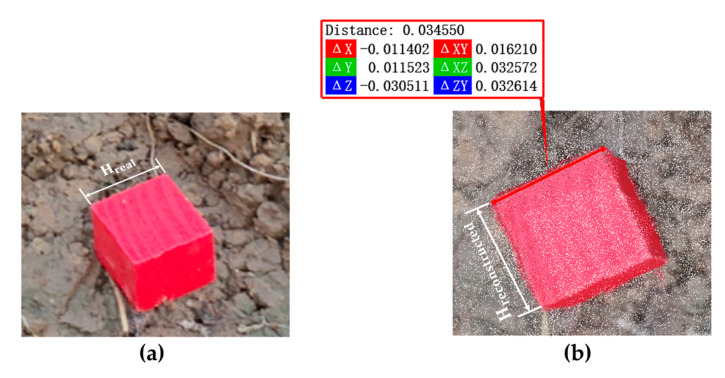
Schematic diagram of the actual dimensions of the calibration block and the point cloud dimensions: (**a**) calibration block; (**b**) calibration block point cloud.

During the data acquisition process, the cubic calibration block and the sesame plant need to be placed together in the scanning scene to ensure that both can be scanned simultaneously and constructed into the same 3D model. Then, the side length of the calibration block in the point cloud model is extracted and compared with its actual physical size to calculate the scale factor between the point cloud and the real world. This scale factor is used for subsequent size correction of various phenotypic parameters of sesame plants to ensure the accuracy of measurement results. The calculation formula for the scaling factor between the real side length and the point cloud side length is shown in Equation (3). A schematic diagram of the actual size and point cloud size of the calibration block is shown in Figure 5.

(3) $$r=\frac{H_{real}}{H_{reconstructed}},$$

In the formula, r is the scaling factor, $H_{real}$ is the actual side length of the calibration block, and $H_{reconstructed}$ is the edge length of the point cloud of the calibration block.

### 2.4. Sesame 3D Point Cloud Organ Segmentation

During the plant growth process, stems are prone to bending or even lodging due to their slenderness [37]. Meanwhile, the number of leaves increases sharply, with sesame (*Sesamum indicum* L.) leaves overlapping and interleaving with each other. Petioles are hidden in the gaps between leaves, and stems are often partially occluded, resulting in blurred and indistinct boundaries of various parts. This makes it difficult for methods based on fixed rules (such as clustering or model fitting) to achieve effective segmentation.

Therefore, this study proposes a sesame plant organ segmentation method based on CAVF-PointNet++ and geometric clustering. Based on the morphological structure of sesame plants, this study proposes the CAVF-PointNet++ model on the basis of the PointNet++ network architecture [38]. By introducing the Context Anchor Attention (CAA) [39] and the Varifocal Loss (VFL) [40] function, the model segments stems, petioles, and leaves, and then uses the curvature-density clustering technique to achieve segmentation between different leaves.

#### 2.4.1. CAVF-PointNet++ Network Architecture

**Figure 6:**
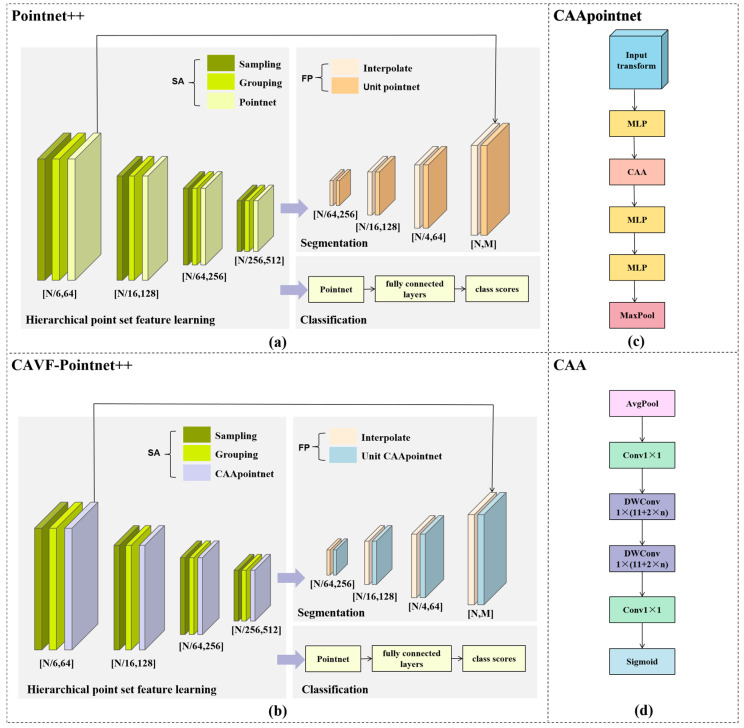
Network architectures of original PointNet++ and improved CAVF-PointNet++: (**a**) PointNet++ network structure; (**b**) CAVF-PointNet++ network structure; (**c**) schematic diagram of CAApoint module structure; (**d**) schematic diagram of CAA module structure.

PointNet++ is an extension of PointNet, designed to more effectively process 3D point cloud data, especially in capturing local geometric structures and handling changes in point cloud density [41]. It mainly introduces a hierarchical feature learning mechanism, which gradually extracts feature representations from local to global, greatly improving the generalization ability and robustness of the model. Its network structure is shown in Figure 6a.

The core idea of PointNet++ lies in realizing hierarchical feature extraction from local to global through stacked Set Abstraction (SA) modules. Each SA module consists of three key steps: first, a Farthest Point Sampling (Sampling) strategy is adopted to determine representative center points; second, spherical neighborhood queries (Grouping) are used to construct a local neighborhood for each center point; finally, a small PointNet network is employed to perform feature encoding on the local point set within the neighborhood.

In addition, to effectively address the issue of uneven point cloud density, PointNet++ further proposes the Multi-Scale Grouping (MSG) and Multi-Resolution Grouping (MRG) mechanisms. Among them, MSG uses spherical neighborhoods of different scales at the same center point for multi-scale feature extraction. It then concatenates these features, boosting robustness to both sparse and dense regions in the point cloud. In contrast, MRG fuses abstract features of sub-regions with the original point features of this layer. This achieves an effective balance between computational efficiency and density adaptability. Meanwhile, to obtain fine point-wise feature representations of the original point cloud for tasks such as point cloud segmentation, PointNet++ also introduces the Feature Propagation (FP) module. Through distance-based interpolation and cross-layer connection mechanisms, this module recovers and propagates high-level abstract features to the original point set.

Due to the similar morphology and severe spatial overlap of different organs such as stems, petioles, and leaves in sesame (*Sesamum indicum* L.) point cloud data, the traditional PointNet++ model has certain limitations in capturing local detail differences and addressing class imbalance issues. To this end, this study proposes the CAVF-PointNet++ model based on PointNet++, and its structure is shown in Figure 6b. By introducing the Context Anchor Attention (CAA) mechanism into the small PointNet network, a CAApoint module is designed. This module adaptively captures local multi-scale contextual information and accurately characterizes the contextual dependencies in point cloud data, thereby enhancing the model’s ability to perceive spatial feature differences and improving the discrimination accuracy between stems, petioles, and leaves. Its structure is shown in Figure 6c.

In addition, by introducing the VFL function, the problem of inter-class imbalance is further alleviated, enabling the model to focus more on hard-to-distinguish samples in confusing classes during training, thus further improving the segmentation performance for fine structures in sesame point cloud data.

The CAA mechanism aims to capture local contextual information by introducing context anchors, thereby enhancing the network’s ability to perceive and represent spatial features. Specifically, this mechanism first performs a Global Average Pooling (AvgPool) operation on the input features to effectively aggregate spatial dimension information and form an initial local context representation. Subsequently, Conv 1 × 1 is used to reduce the dimension of the channel so as to obtain more representative refined context features. Then, by applying Depthwise Separable Convolution (DWConv) along the horizontal and vertical directions, respectively, direction-sensitive spatial context information is extracted to obtain both horizontal and vertical features. Finally, Conv 1 × 1 is used to fuse features from different directions, and an adaptive attention weight is generated through the Sigmoid activation function to accurately capture subtle differences between spatial regions. This effectively improves the network’s performance in tasks of spatial detail feature extraction and segmentation of morphologically similar structures. Its structure is shown in Figure 6d.

This study introduces the VFL function to address the problem of point cloud sample imbalance, which is proposed based on the focal loss function. The core of focal loss lies in introducing a focusing factor to dynamically adjust easy and hard samples: by suppressing the loss contribution of easily classified samples and enhancing the gradient signal of hard-to-classify samples, it guides the model to focus more on “hard samples” during training. However, this method fails to fully consider the differences in feature distribution between positive and negative samples and adopts the same weight adjustment method for both, which limits its performance in scenarios with class imbalance and significant differences in sample quality. To solve this problem, by jointly modeling category labels and prediction quality, the weight distribution of positive and negative samples in the loss function is dynamically adjusted. This enables the model to pay more attention to high-quality positive samples and hard-to-distinguish regions during training, thereby effectively improving the recognition ability for hard-to-distinguish samples and the overall performance in segmentation tasks. The calculation formula is shown in Equation (4):

(4) $$VFL\left(p,q\right)=-q\log\left(p\right)-{\left(1-q\right)}^{\gamma }\log\left(1-p\right),$$

where in the formula, p represents the confidence that the model predicts as the positive class; q represents the confidence of the target label; γ is the focusing factor.

#### 2.4.2. Plant Coordinate System Calibration

**Figure 7:**
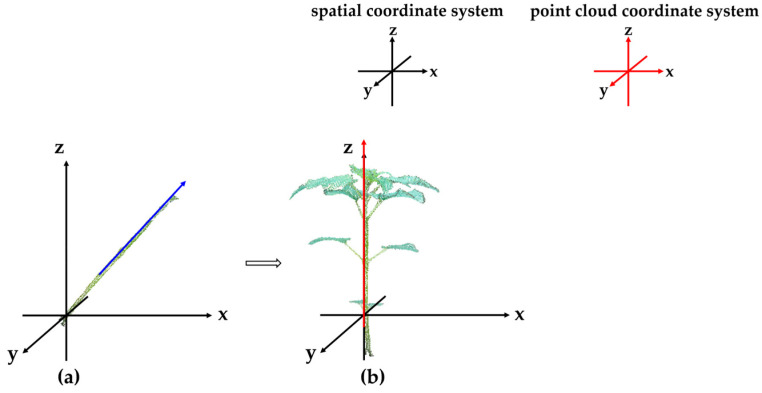
Comparison diagram of coordinate system before and after calibration: (**a**) before coordinate system calibration; (**b**) after coordinate system calibration.

Organ-level semantic segmentation of the 3D point cloud of plants is performed using CAVF-PointNet++ to segment stems, petioles, and leaves. For the segmented stem point cloud data, Principal Component Analysis (PCA) [42] is used to extract the main direction of its morphological features, which is defined as the Z-axis of the spatial coordinate system to facilitate subsequent phenotypic parameter extraction. The coordinate system transformation is shown in Figure 7, where the blue arrow in Figure 7a represents the main morphological direction of the stem point cloud extracted by PCA under the point cloud coordinate system.

#### 2.4.3. Segmentation of Sesame Leaves

To address the problem of severe adhesion at the top of leaves in sesame (*Sesamum indicum* L.) plant point clouds, this paper proposes a method combining curvature threshold and DBSCAN clustering. The point cloud on the surface of a normal leaf is evenly distributed, with gentle changes in the normal vector and small fluctuations in local curvature. In contrast, the adhesion area has a dense and disordered point cloud distribution due to the interweaving and overlapping of point clouds from multiple leaves, resulting in drastic changes in the normal vector and a significant increase in local curvature.

In practical operation, the local geometric curvature of each point in the point cloud is first calculated. This paper adopts a curvature estimation method based on normal vector variation, and the calculation formula is shown in Equation (5):

(5) $$Curvature=\left\|\frac{1}{k}\sum_{i=1}^{k}{\vec{n}}_{i}-{\vec{n}}_{0}\right\|,$$

where in this equation, ${\vec{n}}_{0}$ is the normal vector of the target point, ${\vec{n}}_{i}$ is the normal vector of the i-th point in its neighborhood, and k is the number of points in the neighborhood.

By setting a reasonable curvature threshold, the point cloud of the adhesion area with curvature greater than the threshold is extracted to segment the adhesion area between leaves. Subsequently, the DBSCAN clustering algorithm is applied to the remaining point cloud. Based on the spatial density characteristics of the point cloud, different leaves are divided into independent point cloud clusters, thus completing the overall sesame leaf segmentation.

### 2.5. Phenotypical Parameter Extraction Methods

When conducting phenotypic real data measurement, standardized operating procedures should be strictly followed. Objective and accurate measurement tools and methods should be adopted to minimize the interference of human factors on measurement results. Meanwhile, 3 repeated experiments need to be carried out to ensure the reliability and accuracy of the data. The specific operation methods are as follows:

Plant height: Use a tape measure. Vertically measure from the junction of the sesame (*Sesamum indicum* L.)’s root base and ground to the plant’s highest point in natural growth. Record the straight-line distance.

Stem diameter: Select the stem cross-section 2 cm above ground. The stem here is roughly quadrangular prism-shaped. Use a vernier caliper to measure its diameter along two perpendicular directions. Ensure caliper jaws fit the stem edge. Record the average of the two measurements as stem diameter.

Leaf length: Align the tape measure’s zero scale with the leaf base. Measure the linear distance along the leaf midrib to the tip. Record this distance as leaf length.

Leaf width: Place the tape measure perpendicular to the leaf midrib. Measure the straight-line distance between edges at the leaf’s widest part. Take this as leaf width.

Leaf angle: Focus on the angle between the petiole and main stem. Align the protractor’s center with their intersection. Place the protractor’s baseline along the main stem. Read the angle corresponding to the petiole as the leaf angle.

Leaf area: Place the leaf with a 2 cm-side cube wooden block on a black backdrop. Take a photo. To improve accuracy, flatten the leaf before shooting to reduce errors from curling or warping. Binarize the acquired image. Convert the leaf’s actual area using the ratio of the wooden block’s real area to its pixel area in the image.

Unlike manual measurement, the acquisition of point cloud phenotypic parameters relies on 3D point cloud models. It enables non-destructive, automated, and high-throughput measurement while ensuring plant integrity. The specific methods are as follows:

Plant height and leaf width: Measured by constructing an Axis-Aligned Bounding Box (AABB) [43]. Plant height is the extreme difference in the bounding box along the Z-axis, and leaf width is the extreme difference along the X-axis.

Stem diameter: Measured by extracting a section of the point cloud 2 cm above the ground. First, project this section of the point cloud onto the horizontal XY plane and extract the cross-sectional contour of the stem at this height. Then, perform circle fitting on these projected points, and use the diameter of the fitted circle as the estimated value of the stem diameter.

Leaf length: Extract the two farthest points in the leaf point cloud as the start and end points. Between them, gradually select the point closest to the direction of the end point along the middle of the leaf to construct a path approximating the leaf midrib. Finally, accumulate the length of this path as the estimated leaf length.

Leaf angle: Leaf angle refers to the angle between the petiole and the stem. Using PCA to determine the vector $\vec{b}$ of the petiole and defining the Z-axis direction as the vector $\vec{a}$, the leaf angle is obtained by calculating the angle between these two vectors. The calculation formula is shown in Equation (6):

(6) $$\left\{\begin{array}{c} \left\|\vec{a}\right\|=\sqrt{a_{1}^{2}+a_{2}^{2}+a_{3}^{2}}\\ cos\theta =\frac{\vec{a}.\vec{b}}{\left\|\vec{a}\right\|\left\|\vec{b}\right\|} \end{array}\right.,$$

where $\left|\vec{a}\right|$ and $\left|\vec{b}\right|$ represent the modulus of $\vec{a}$ and $\vec{b}$, respectively, and $\vec{a}$ = ($a_{1},a_{2},a_{3}$).

Leaf area: To improve the efficiency and accuracy of leaf area calculation, the input 3D point cloud data is first projected onto the principal component plane using the PCA method. Then, the Delaunay triangulation method [44] is used to construct a planar triangular mesh for the projected points. The calculation formula for the total area is shown in Equation (7):

(7) $$S_{total}=\sum_{k=1}^{m}S_{k}=\frac{1}{2}\sum_{k=1}^{m}\left||\vec{v_{1k}}\times \vec{v_{2k}}|\right|,$$

where $S_{total}$ represents the total area, m represents the number of triangles, and $\vec{v_{1k}}$ and $\vec{v_{2k}}$ are the vectors corresponding to the two sides of the triangle.

**Figure 8:**
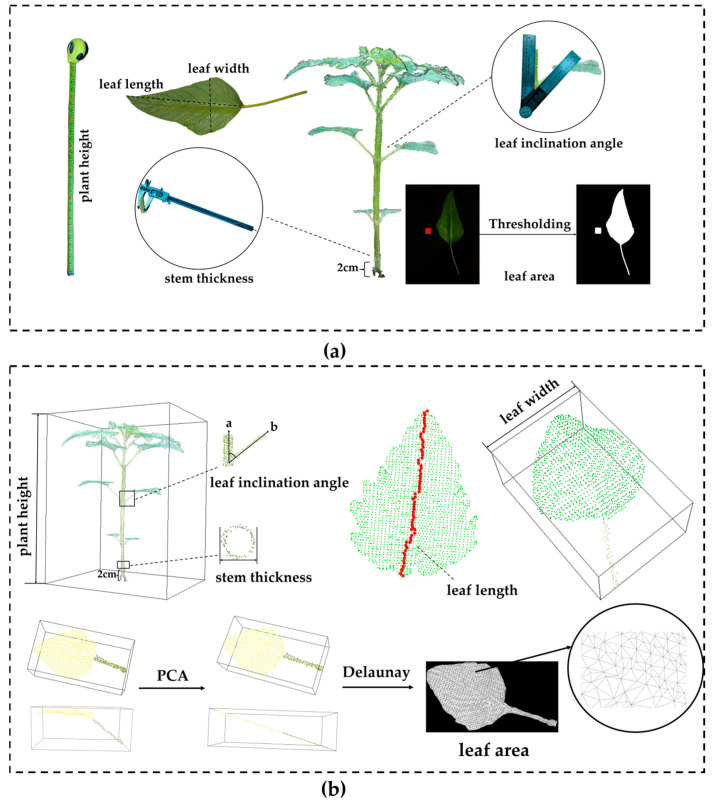
Schematic diagram of phenotypic parameter acquisition methods: (**a**) acquisition method of true values of phenotypic parameters; (**b**) schematic diagram of point cloud phenotypic parameter extraction.

To intuitively demonstrate the acquisition method of phenotypic parameters, Figure 8 presents the acquisition method of the true values of phenotypic parameters and the extraction method of point cloud phenotypic parameters, clearly showing the measurement method of each phenotypic parameter in a graphical form.

## 3. Results

### 3.1. Dataset Annotation and Model Training

In this study, the collected high-precision 3D point cloud data of 36 different morphological features were used as the dataset, covering typical growth stages from the seedling stage to the mature stage. Data annotation was carried out based on CloudCompare, a professional point cloud processing software with efficient point cloud segmentation and label assignment functions. The annotated point clouds were divided into 3 categories: the first category is sesame (*Sesamum indicum* L.) stems, assigned label 0; the second category is sesame petioles, assigned label 1; and the third category is sesame leaves, assigned label 2. The annotated sesame point clouds were saved in TXT file format and divided into training, validation, and test sets in an 8:1:1 ratio.

Model training was conducted on the PyTorch (Version 2.2.1) deep learning framework. The hardware configuration used included a 13th Gen Intel(R) Core(TM) i7-13700KF (3.40 GHz), 64 GB of RAM, and an NVIDIA GeForce RTX 4080 with 16 GB of video memory, running on the Windows 11 operating system. During the training process, the parameters for the number of samples, batch size, decay rate, training epochs, learning rate, and optimizer were set to 10,000, 4, 0.0001, 200, 0.001, and Adam, respectively. In the feature extraction process, we adopted the MSG feature learning strategy to capture local information as comprehensively as possible.

### 3.2. Organ Segmentation Evaluation Metrics

The segmentation of stems, petioles, and leaves uses the mean Intersection over Union (mIoU) and Overall Accuracy (OA) as evaluation metrics for the segmentation task, with the calculation formulas shown in Equations (8) and (9):

(8) $$mIoU=\frac{1}{M}\sum_{i=1}^{M}\frac{TP}{TP+FP+FN},$$

(9) $$OA=\frac{TP+TN}{TP+TN+FP+FN},$$

where in the formula, M represents the segmentation category; False Negative (FN) represents the number of samples classified as negative but are actually positive; True Negative (TN) is the number of samples classified as negative but are actually negative; False Positive (FP) is the number of samples classified as positive but are actually negative; and True Positive (TP) is the number of samples classified as positive and are actually positive.

The effectiveness of segmentation between different leaves is evaluated using three metrics: precision (P), recall (R), and F1-score ($F_{1}$). The formulas are shown in Equations (10)–(12):

(10) $$P=\frac{TP}{TP+FP},$$

(11) $$R=\frac{TP}{TP+FN},$$

(12) $$F_{1}=2\cdot \frac{P\cdot R}{P+R},$$

where TP represents the actual points predicted as leaf A, FP represents the actual points that are not leaf A but are incorrectly classified as leaf A, and FN represents the actual points that are leaf A but are incorrectly classified as other leaves.

To assess the consistency between the extracted phenotypic parameters and the manually measured results, a linear regression analysis was used to compare the two, and the accuracy and error were quantified using the coefficient of determination (R) and the Root Mean Square Error (RMSE). The calculation formulas are shown in Equations (13) and (14):

(13) $$R^{2}=1-\frac{\sum_{i=1}^{n}{\left(y_{i}-{\hat{y}}_{i}\right)}^{2}}{\sum_{i=1}^{n}{\left(y_{i}-\bar{y}\right)}^{2}},$$

(14) $$RMSE=\sqrt{\frac{1}{n}\sum_{i=1}^{n}{\left(y_{i}-{\hat{y}}_{i}\right)}^{2}},$$

where in the equations, $y_{i}$ is the actual value, ${\hat{y}}_{i}$ is the predicted value, $\bar{y}$ is the average value of the actual values, and n represents the number of samples.

### 3.3. Ablation Experiment

To verify the effectiveness of each improved module in the proposed CAVF-PointNet++ model, a systematic ablation experiment was designed and conducted. The experiment evaluated the performance improvement effect of introducing a single module and combined modules on the model in the fine structure segmentation task of sesame (*Sesamum indicum* L.) point cloud data, thereby clarifying the independent and synergistic effects of each module in performance improvement. The experimental results are shown in Table 1.

As can be seen from the experimental results, the unmodified PointNet++ network achieved OA and mIoU of 0.9521 and 0.7892, respectively, on the sesame point cloud dataset, serving as the baseline for performance comparison. After introducing the CAA module alone, the OA improved to 0.9612 and the mIoU improved to 0.8018, significantly outperforming the baseline performance. This indicates that the CAA module effectively enhances the network’s ability to perceive and distinguish local fine structures in point clouds by capturing richer contextual dependencies. In addition, using the VFL function alone also achieved certain performance gains, with OA improving to 0.9586 and mIoU improving to 0.7928, indicating that VFL has a positive effect on optimizing category weight distribution and mitigating the adverse effects of category imbalance.

**Figure 9:**
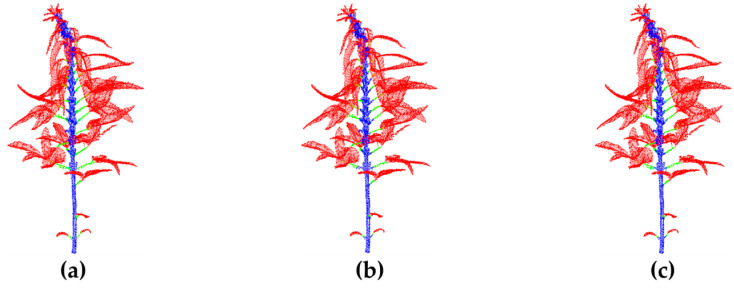
Comparison of segmentation results of different models on the sesame 3D point cloud: (**a**) manually annotated results; (**b**) segmentation results of the original PointNet++; (**c**) segmentation results of CAVF-PointNet++.

When both CAA and VFL are introduced as improvement strategies, the model achieves optimal performance, with an OA of 0.9693 and a mIoU of 0.8256, representing improvements of 1.72% and 3.64% over the baseline model, respectively. This result fully demonstrates the excellent synergy between CAA and VFL in feature extraction and loss function optimization. CAA effectively enhances the expression ability of point cloud features, while VFL enhances the network’s ability to distinguish between easily confused categories by dynamically optimizing the training weights of difficult and easy samples, thereby further improving the model’s segmentation accuracy of fine structures in sesame point cloud data. Figure 9 shows the segmentation results of the CAVF-PointNet++ network using the unmodified PointNet++ network and the CAVF-PointNet++ network with both CAA and VFL improvements.

As can be clearly seen from Figure 9b, although the original PointNet++ model can roughly identify and segment the main structures of the plant, such as stems, petioles, and leaves, it still exhibits misclassification in some details and boundary regions, particularly at the junctions between stems and petioles or in complex, intersecting areas. The classification accuracy and boundary clarity are suboptimal, highlighting the limitations of the original PointNet++ model in capturing local details and contextual information. In comparison, the CAVF-PointNet++ model proposed in this paper significantly improves the above shortcomings by introducing the CAA mechanism and the VFL function. From the segmentation results in Figure 9c, it can be seen that the model distinguishes the details and boundaries of the plant point cloud data more accurately and clearly, especially in complex areas where stems and petioles intersect, achieving more accurate and refined structural segmentation. This indicates that the CAA module can effectively enhance the model’s perception of local details and spatial context information in point clouds, while VFL further strengthens the model’s focus on and discrimination ability for difficult categories. The two work together synergistically, enabling the improved model to exhibit better generalization performance and robustness. This also fully verifies the effectiveness and advantages of the proposed improvement method in the sesame point cloud segmentation task.

### 3.4. The Influence of Parameters on the Segmentation Accuracy of Different Leaves

When using the curvature-density clustering method to segment point clouds of sesame (*Sesamum indicum* L.) plant leaves, the key parameters include the local curvature threshold (Threshold), neighborhood radius (ε), and minimum number of neighborhood points (MinPts). These parameters have a significant impact on the segmentation results. Reasonable parameter settings can effectively improve the accuracy of leaf segmentation, while incorrect or unreasonable settings may lead to poor segmentation results or the appearance of a large number of noise points.

**Figure 10:**
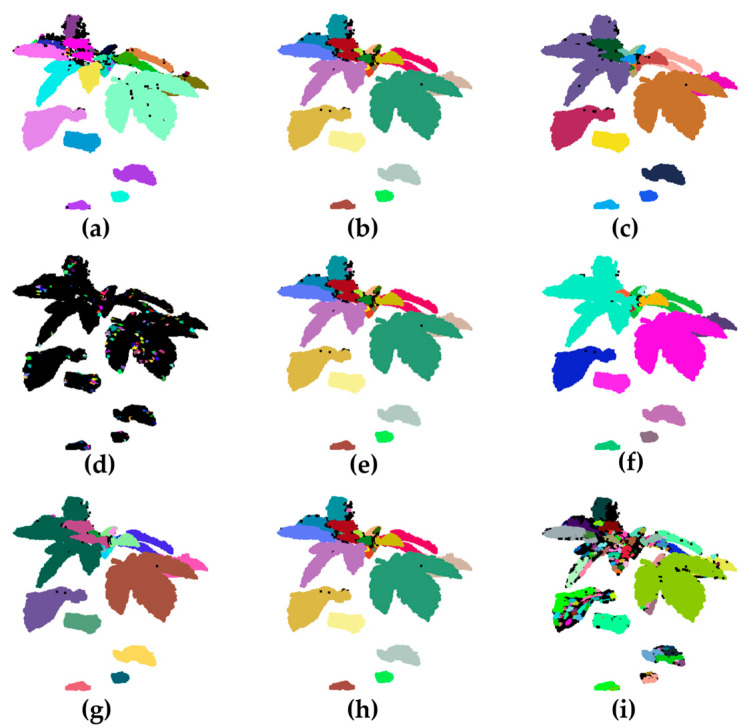
Effect of different parameters on the segmentation of sesame leaves: (**a**) Threshold = 0.1, ε = 0.006, MinPts = 10; (**b**) Threshold = 0.2, ε = 0.006, MinPts = 10; (**c**) Threshold = 0.3, ε = 0.006, MinPts = 10; (**d**) Threshold = 0.2, ε = 0.004, MinPts = 10; (**e**) Threshold = 0.2, ε = 0.006, MinPts = 10; (**f**) Threshold = 0.2, ε = 0.008, MinPts = 10; (**g**) Threshold = 0.2, ε = 0.006, MinPts = 5; (**h**) Threshold = 0.2, ε = 0.006, MinPts = 10; (**i**) Threshold = 0.2, ε = 0.006, MinPts = 15.

To quantitatively analyze the specific effects of each parameter on the leaf segmentation performance, Figure 10 shows the changes in segmentation performance when the three key parameters are adjusted individually. By comparing the results, one can directly observe the specific effects of different parameter combinations on the point cloud segmentation of sesame plant leaves. In this method, points with curvature greater than a set threshold are defined as contact areas and removed from the original point cloud. The remaining point cloud is segmented into leaves using the DBSCAN clustering method. In the clustering results, black points represent noise points that have not been assigned to any valid cluster.

Figure 10a–c show the changes in the leaf segmentation effect as the curvature threshold is gradually increased under the conditions of ε set to 0.006 and MinPts set to 10. In Figure 10a, the Threshold is set to 0.1. Setting a smaller curvature threshold can identify a large number of high-curvature points, which effectively block the connection between adhered leaves and improve segmentation accuracy. However, at the same time, subtle undulations in the point cloud are also misidentified as contact areas, causing some of the actual leaf edges to be cut incorrectly. Figure 10b shows that with a Threshold set to 0.2, the number of contact points is moderate, which can remove most of the leaf junctions while preserving the integrity of the leaf structure. At this point, there is an appropriate amount of noise, and the leaves are clearly divided, which is a relatively balanced parameter setting. Figure 10c, with the Threshold set to 0.3, when the threshold is further increased, only a small number of contact areas are detected. Due to the lack of effective boundaries, multiple leaves are incorrectly classified into the same cluster during clustering, resulting in obvious adhesion.

Figure 10d–f show the effect of different neighborhood radii on the clustering results under the conditions of Threshold set to 0.2 and MinPts set to 10. In Figure 10d, ε is set to 0.004. A smaller neighborhood radius causes each cluster to accommodate only points that are very close to each other, resulting in the same sesame leaf being divided into multiple small clusters, which clearly shows the phenomenon of “over segmentation.” In Figure 10e, ε is set to 0.006, and a medium-sized neighborhood radius allows the algorithm to divide each leaf more reasonably. Most leaves are accurately identified as independent clusters, and edge details are well preserved, resulting in good overall clustering results. In Figure 10f, ε is set to 0.008. The excessive neighborhood radius allows the distances between different leaves to be merged, resulting in obvious “under-segmentation” and multiple leaves being misjudged as a single cluster.

Figure 10g–i show the effect of the number of minimum neighboring points on the clustering results under the conditions of Threshold set to 0.2 and ε set to 0.006. In Figure 10g, MinPts is set to 5, and the algorithm has low requirements for local point density. Low-density areas are also easily classified into clusters, resulting in ineffective differentiation between some leaves and obvious leaf adhesion. In Figure 10h, MinPts is set to 10, the number of noise points is moderate, and noise and adhesion phenomena are balanced, resulting in the best overall segmentation effect. In Figure 10i, MinPts is set to 15, and the density criterion is further increased. Only high-density regions can form clusters, and some valid data are misjudged as noise, resulting in a significant increase in the number of noise points. A large number of small, isolated clusters appear in the point cloud, affecting the overall segmentation integrity.

Comprehensive analysis of the effects of various parameters on the leaf segmentation effect shows that when Threshold is set to 0.2, ε is set to 0.006, and MinPts is set to 10, the leaves are segmented most accurately, and the clustering results are clear and stable.

**Figure 11:**
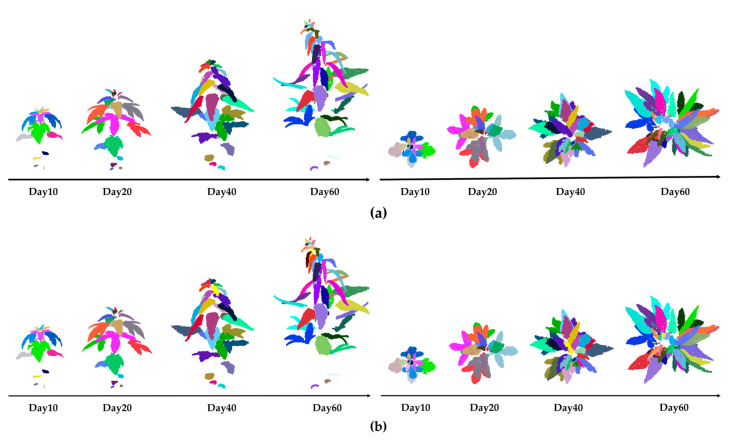
Comparison of sesame leaf segmentation results at different growth days: (**a**) algorithm-based segmentation results; (**b**) manual segmentation results.

In this study, automated segmentation of sesame plant leaves was performed on the 10th, 20th, 40th, and 60th days after transplantation. To verify the reliability of the algorithm’s segmentation effect, CloudCompare software was used for manual fine segmentation of the plant point clouds at the same time points, which served as the comparison benchmark. The algorithm-based segmentation results of plant point clouds at different growth stages are shown in Figure 11a (left: front view; right: top view), and the corresponding manual segmentation results are shown in Figure 11b (left: front view; right: top view).

**Figure 12:**
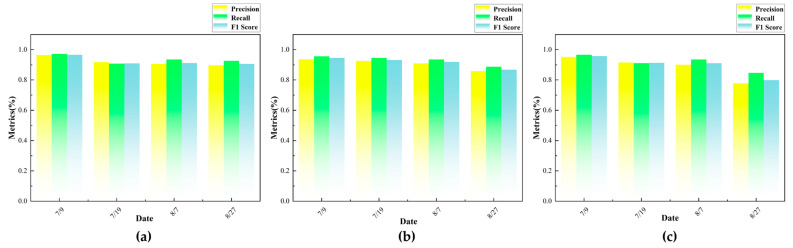
Comparison of leaf segmentation performance of different sesame varieties at different time points after transplanting: (**a**) Dongbao No.2; (**b**) Fenzhi No.2; (**c**) Fenzhi No.13.

The leaf segmentation performance metrics for a total of 36 point cloud data samples from three sesame varieties at four time points after transplantation are shown in Figure 12. It can be seen that the segmentation performance of each variety shows a relatively consistent trend over time. From all indicators, with the increase in time, P, R, and $F_{1}$ show a trend of relative stability or slight decline at first, followed by a significant decline at 60 days after planting. In the early stages after planting, sesame plants are in their initial growth phase, with relatively few leaves and little overlap between them. This makes it relatively easy for the segmentation model to identify and segment leaves, resulting in relatively stable P, R, and $F_{1}$ values overall. As the plants grow, the number of leaves increases, and overlapping and occlusion between leaves become more frequent, especially in the densely packed upper leaves, leading to blurred boundaries and making it difficult for the model to accurately segment the leaves. As a result, the accuracy of P, R, and $F_{1}$ significantly decreases.

### 3.5. Correlation Analysis Between Measured Values and Actual Values of Phenotypic Parameters

**Figure 13:**
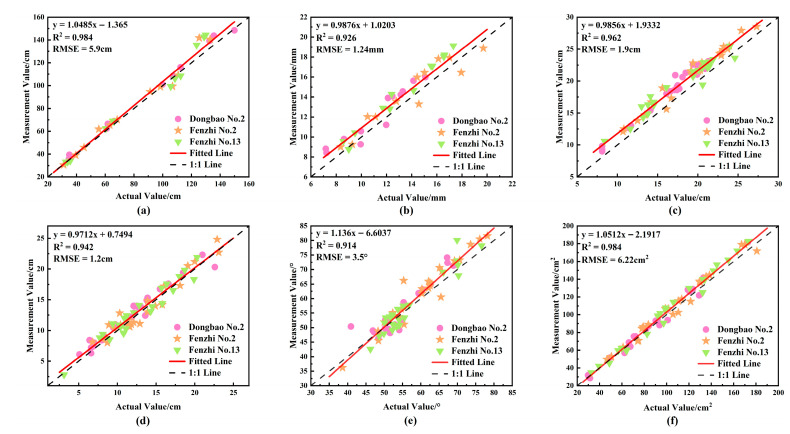
Correlation analysis between actual values and measured values of phenotypic parameters: (**a**) plant height; (**b**) stem diameter; (**c**) leaf length; (**d**) leaf width; (**e**) leaf angle; (**f**) leaf area.

The results of the correlation analysis between the measured values and actual values of phenotypic parameters of three sesame (*Sesamum indicum* L.) varieties are shown in Figure 13. For plant height, the *R*^2^ and RMSE are 0.984 and 5.9 cm, respectively; for stem diameter, the *R*^2^ and RMSE are 0.926 and 1.24 mm, respectively; for leaf length, the *R*^2^ and RMSE are 0.962 and 1.9 cm, respectively; for leaf width, the *R*^2^ and RMSE are 0.942 and 1.2 cm, respectively; for leaf angle, the *R*^2^ and RMSE are 0.914 and 3.5°, respectively; and for leaf area, the *R*^2^ and RMSE are 0.984 and 6.22 cm^2^, respectively.

The results of the correlation analysis on six phenotypic parameters—including plant height, stem diameter, leaf length, leaf width, leaf angle, and leaf area—of three sesame varieties (Dongbao No.2, Fenzhi No.2, and Fenzhi No.13) showed that the fitted lines were very close to the ideal relationship y = x, with all *R*^2^ values exceeding 0.9. This indicates a good linear consistency between the point cloud measured values and the actual values, verifying the high accuracy of the automatic extraction method for each parameter.

Among them, plant height and leaf area had the highest correlation, demonstrating that the extreme difference of extraction based on the AABB method and the image binarization conversion method can effectively capture features such as the overall plant height and leaf area. Leaf length and leaf width also exhibited good fitting effects; the adopted measurement method based on midrib path extraction and extreme value envelopment could accurately characterize the geometric morphological features and spatial expansion trends of leaves. The *R*^2^ for stem diameter was 0.926, which, although slightly lower, still fell within the acceptable range. The error mainly stemmed from the fitting deviation introduced by simplifying the irregularly shaped cross-section into a circle. The *R*^2^ for leaf angle was 0.914, the lowest among the six parameters. This might be due to the fact that the petiole is not an ideal straight structure but has slight twists or bends, leading to deviations in angle estimation; however, the overall trend was still consistent with the actual measurements.

In general, the point cloud phenotypic measurement method constructed in this study not only demonstrates high precision and stability in measuring the main morphological phenotypes of sesame but also shows good performance in multi-variety applicability, providing reliable technical support for sesame phenotypic research and high-throughput data acquisition.

## 4. Discussion

This study proposes a method for automatic segmentation of sesame (*Sesamum indicum* L.) plant organs. It also enables the extraction of phenotypic parameters. The method is based on CAVF-PointNet++ and geometric clustering. Through the analysis of six indicators—plant height, stem diameter, leaf length, leaf width, leaf angle, and leaf area—and comparative analysis with actual values, it is found that the *R*^2^ of key parameters all exceed 0.90 and the RMSE is within a small range. This indicates that the method has high accuracy in extracting these phenotypic parameters and can well reflect the actual phenotypic characteristics of plants. Given the excellent performance of this method in phenotypic parameter extraction in the field of sesame breeding, breeders can use this method to quickly and accurately obtain phenotypic parameters of a large number of sesame plants. By analyzing and comparing the phenotypic characteristics of plants of different varieties or under different growth conditions, varieties with excellent phenotypic traits can be screened out, thereby accelerating the breeding process and improving breeding efficiency.

Among the specific parameters, the measurement accuracy of leaf angle and stem diameter is relatively slightly lower. The estimation of leaf angle relies on PCA to extract the petiole direction, which is prone to deviate from the real posture when the point cloud is sparse or the petiole is curved; meanwhile, fixing the Z-axis as the stem direction may also introduce errors. In the future, robustness can be improved through RANSAC fitting and multi-point angle averaging. Stem diameter measurement is limited by the stability of the circle fitting method when the cross-section is irregular or the plant is tilted; in subsequent work, ellipse fitting and multi-height cross-section averaging can be attempted to enhance accuracy.

The combination of OpenMVG + OpenMVS was used for the 3D reconstruction of sesame plants. Although this approach has achieved the construction of the overall morphological structure of the plants to a certain extent, it also reveals relatively obvious limitations. In the reconstruction results, small fruits such as sesame seeds cannot be clearly presented, which directly makes it impossible to accurately count the number of fruits. To address this issue, subsequent studies plan to introduce a depth camera for 3D reconstruction. Compared with image-matching-based reconstruction methods, depth cameras have a stronger ability to capture details of tiny objects. This is expected to solve the problems that the current reconstruction method cannot clearly present sesame seeds and cannot count the number of fruits, thereby providing more comprehensive and accurate data support for the phenotypic analysis of sesame plants.

It should be noted that the model in this study was only tested on three sesame varieties (Dongbao No.2, Fenzhi No.2, and Fenzhi No.13, totaling nine plants with 36 different morphological characteristics at four time points) in a single experimental area (Institute of Economic Crops, Shanxi Agricultural University, Fenyang City, Lüliang City, Shanxi Province). Its applicability across a wider range of varieties and diverse environmental conditions still needs further verification. Future research will expand the variety scope and sampling areas to enhance the universality and robustness of the method. Furthermore, the method proposed in this study can also be combined with RGB imaging technology mounted on mobile platforms (such as unmanned aerial vehicles and ground robots) for large-scale field high-throughput phenotyping, thereby expanding its application prospects in practical production and crop phenotypic research. Meanwhile, if organ-scale geometric structure parameters can be combined with narrow-band or RGB reflectance indices, it is expected to achieve a more comprehensive characterization of sesame phenotypes.

## 5. Conclusions

The organ segmentation method proposed in this paper was tested on sesame (*Sesamum indicum* L.) plants with different growth days. In the stem, petiole, and leaf segmentation tasks, the OA of this method reached 96.93% and the mIoU was 82.56%. Compared with the results of manual leaf segmentation, the P, R, and $F_{1}$ obtained by the clustering method were all in the higher range, indicating a better segmentation effect. The experimental results above indicate that the method proposed in this study can accurately segment sesame organs.

The average running time of the algorithm used in this study is 23.21 min. It can complete tasks such as point cloud reconstruction, organ segmentation, and phenotypic extraction in a relatively short time, thus demonstrating high timeliness.

The point cloud and deep learning-based system adopted in this study can quickly, accurately, and multi-dimensionally acquire plant morphological information without interfering with the normal growth of plants. Compared with traditional manual measurement (or measurement with handheld tools), this method not only reduces reliance on manual operations but also yields highly reliable measurement results, as the *R*^2^ values of all six phenotypic parameters exceed 0.90 and the RMSE values are within a small range.

In summary, the method proposed in this study can accurately and efficiently complete the tasks of organ segmentation and phenotypic extraction of sesame plants. It can provide strong support for sesame growth research and variety breeding, reduce research costs, and is of great significance for promoting the automation of sesame phenotypic analysis and the development of smart agricultural technologies.

## Figures and Tables

**Table 1:** Results of ablation experiments.

PointNet++	CAA	VFL	*OA*	*mIoU*
√	×	×	0.9521	0.7892
√	√	×	0.9612	0.8018
√	×	√	0.9586	0.7928
√	√	√	**0.9693**	**0.8256**

Among them, the optimal results are indicated in bold.

## Data Availability

The article contains original data from this study, which should be available upon reasonable request by contacting the corresponding author.
